# Factors Associated with Outcomes of Percutaneous Transluminal Renal Angioplasty in Patients with Renal Artery Stenosis: A Retrospective Analysis of 50 Consecutive Cases

**DOI:** 10.1155/2018/1952685

**Published:** 2018-01-04

**Authors:** Tetsutaro Matayoshi, Kei Kamide, Ryoichi Tanaka, Tetsuya Fukuda, Takeshi Horio, Yoshio Iwashima, Fumiki Yoshihara, Satoko Nakamura, Hajime Nakahama, Yusuke Ohya, Yuhei Kawano

**Affiliations:** ^1^Division of Hypertension and Nephrology, Department of Medicine, National Cardiovascular Center, Osaka, Japan; ^2^Department of Cardiovascular Medicine, Nephrology and Neurology, University of the Ryukyus, Okinawa, Japan; ^3^Division of Health Sciences, Osaka University Graduate School of Medicine, Osaka, Japan; ^4^Department of Radiology, National Cardiovascular Center, Osaka, Japan; ^5^Division of Cardiovascular Radiology, Department of Radiology, Iwate Medical University, Iwate, Japan; ^6^Department of General Internal Medicine 3, Kawasaki Medical School, Okayama, Japan; ^7^Diage Kobe Clinic, Hyogo, Japan; ^8^Department of Medical Technology, Teikyo University, Fukuoka Campus, Fukuoka, Japan

## Abstract

**Background:**

The results of recent trials have brought some confusion to the treatment strategy for renal artery stenosis (RAS). To evaluate the applicability of percutaneous transluminal renal angioplasty (PTRA) for RAS, we extracted the factors that may affect the effectiveness of PTRA from cases experienced at a hypertension center.

**Methods and Results:**

We retrospectively assessed the blood pressure (BP) lowering effects and renoprotective effects in 50 consecutive patients that had hemodynamically significant RAS and had undergone PTRA and stenting during 2001–2005. Subjects were diagnosed with atherosclerotic RAS (42), fibromuscular dysplasia (6), or Takayasu disease (2). After PTRA, BP significantly lowered from 152.3/80.3 mmHg to 132.6/73.2 mmHg (*p* < 0.05), but the estimated glomerular filtration rate (eGFR) did not change significantly. There were no factors associated with the BP lowering effects of PTRA. The baseline resistive index (RI) was negatively correlated with the change in eGFR (*p* < 0.05). After correction for age, sex, BMI, and the dose of contrast medium, the association of RI with change in eGFR remained significant.

**Conclusion:**

In cases with hemodynamically significant RAS, PTRA lowered BP but was not effective in improving renal function. Higher baseline RI may be a factor for predicting poor clinical course of renal function after PTRA.

## 1. Introduction

Renal artery stenosis (RAS) is a cause of renovascular hypertension (RVHT) and ischemic nephropathy and has been demonstrated to be a predictor of future cardiovascular events [1]. Percutaneous transluminal renal angioplasty (PTRA) has been one of the common treatments for RAS despite recent developments in therapeutics [2, 3]. Using retrospective data, Bonelli et al. reported that 60–90% of 320 RAS patients who underwent PTRA exhibited some benefits after PTRA [4]. The efficacy of PTRA for the treatment of RVHT differs among causes of RAS, such as atherosclerosis, fibromuscular dysplasia (FMD), and Takayasu arteritis, as well as with or without stent placement. BP lowering effects with a high hypertension cure rate by PTRA are reportedly better in FMD than in atherosclerosis [4]. Cure or improvement rates of RVHT after PTRA are reported to be 8 or 70–76% in atherosclerotic stenosis and 22–24% or 63% in FMD cases, respectively [4–6]. However, most trials failed to demonstrate significant improvement in renal function after PTRA [5].

Two large-scale randomized controlled clinical trials (RCTs), ASTRAL (Angioplasty and Stent for Renal Artery Lesions) trial [7], and CORAL (Cardiovascular Outcomes in Renal Atherosclerotic Lesions) trial [8] compared PTRA and conventional medication therapy regarding outcomes for BP lowering effects, renal protection (in ASTRAL), and onset of future cardiovascular events (in CORAL). Both studies could not demonstrate any advantages of PTRA compared with conventional medication therapy. STAR (Stent Placement in Patients With Atherosclerotic Renal Artery Stenosis and Impaired Renal Function), an RCT, which enrolled a relatively small number of patients, also could not demonstrate the renoprotective effects of PTRA in patients with RAS and impaired renal function [9]. Consequently, PTRA for RAS has become less recommended [10].

The current guidelines for atherosclerotic RAS recommend PTRA for patients with hemodynamically significant RAS or with the following conditions: flash pulmonary edema, rapidly declining renal function or refractory hypertension [11–15]. Such patients were excluded from ASTRAL and CORAL.

After ASTRAL and CORAL, physicians, such as cardiologists or radiologists, who handle RAS tend to hesitate to perform PTRA. However, recent recommendations and review articles have demonstrated the importance of PTRA [16, 17]. Therefore, therapeutic strategies to treat RAS are confused, and more information about patient selection is needed to perform PTRA. Several reports have clarified the determining factors for PTRA efficacy [18, 19]. Radermacher et al. has reported that a resistive index (RI) > 0.80 evaluated by renal Doppler ultrasonography (RDU) predicted outcomes in BP and renal function after PTRA in patients with RAS whose RA stenosis was over 50% [18]. Therefore, PTRA may be an effective therapy for patients with RAS if it could be performed for patients selected by RI with RDU. As patients with RAS have several features which may affect the prognosis of hypertension and renoprotection, such as the pathogenesis of atherosclerotic RAS, presence of dyslipidemia, diabetes mellitus (DM), renal impairment or renal failure, and their atherosclerotic complications of other arteries [20], such conditions should be considered when making strategies for RAS.

There are few reports on the effectiveness of PTRA, and there are even fewer reports of the Japanese or Asian patients. The aim of this study was to clarify factors associated with outcomes in PTRA for RAS patients with hemodynamically significant stenosis, before ASTRAL and CORAL, to reconsider the effectiveness of PTRA for RAS patients.

## 2. Subjects and Methods

We retrospectively assessed the influences of several factors on clinical courses of patients who had undergone PTRA. The subjects consisted of 50 consecutive patients who had their first PTRA between January 2001 and September 2005 in the National Cardiovascular Research Center in Osaka, Japan. Patients who had undergone two or more rounds of PTRA for restenosis of the treated lesion or progression of opposite-side RAS were excluded. PTRA was indicated for hemodynamically significant RAS with either of the following: (1) peak systolic velocity ≥ 1.8 m/sec by RDU, (2) diameter or area stenosis rate ≥ 75% by magnetic resonance imaging angiography, and (3) prolonged vascular, functional, and distribution phases by renogram. Cases without viability of the affected kidneys (RI evaluated by RDU < 0.8 and/or low distribution of the affected kidney evaluated by renogram) were excluded. Renal artery stenting was done in all cases at the ostial and proximal lesion without distal protection or filtration device. The clinical courses after PTRA in our institute were good overall. Major complications, such as cholesterol crystal embolism, dissection of the renal artery, or other vascular disease, were not observed in the present study subjects.

BP was measured on the days before PTRA (w0) and 1-2 weeks after PTRA (w1). Serum creatinine levels (S-Cre: enzymatic method) were obtained at the day before PTRA (W0), 1-2 weeks after (w1), and 1 year (+/−4 weeks) after PTRA (y1).

Primarily, we compared the change in mean blood pressure (MBP), use of antihypertensive drugs, and estimated glomerular filtration rate (eGFR) between before and after PTRA.

MBP was calculated as follows:

(1) $$\begin{array}{c} MBP=\frac{\left(\text{Systolic BP}+\text{Diastolic BP}\times 2\right)}{3}. \end{array}$$

eGFR was calculated by the following formula:

(2) eGFR=194×S-Cre−1.094×age−0.287  if  female  subjects,×0.739.

Successful BP reduction was defined as a 5 mmHg or more reduction in w1 mean BP compared with the measurement at w0 or reduction in the dosage of one or more antihypertensive medicines. Course of renal function (eGFR) was stratified into “not changed or improved” (w0 ≥ y1) or “worsened” (w0 < y1).

Additionally, we assessed influences on the BP lowering and renoprotective effects of PTRA by several confounding factors, including age, sex, body mass index (BMI), presence of bilateral stenosis, RI in the affected kidney, impaired glucose tolerance (IGT), DM (DM: expressing DM pattern in oral glycemic tolerance test or currently prescribed hypoglycemic agent), dyslipidemia, RI: (1 – (end diastolic velocity/peak systolic velocity)) [18], plasma renin activity (PRA), MBP (w0) eGFR (<60 mL/min/1.73 m^2^), and the dose of contrast medium by univariate and multivariate analysis. We adapted common log conversion of eGFR and PRA for analysis because they did not follow a standard normal distribution.

All data were analyzed using JMP 9 software (SAS Institute Inc., Cary NC US). All continuous values were expressed as the mean +/− SD and categorical variables were reported as percentages. BP and eGFR were examined. The time courses of BP and eGFR were examined using the paired *t*-test. Between the groups of the BP lowering effects, categorical variables were compared by *χ*^2^ analysis, and continuous variables were compared by unpaired *t*-test. The influence of each parameter on the regression of eGFR was analyzed by unpaired *t*-test or linear regression analysis. *p* values < 0.05 were considered as statistical significant.

## 3. Results

The characteristics of the 50 patients (male 37, female 13) were as follows. All patients were East Asians. Flash pulmonary edema or rapidly declining renal function was not observed on or before admission. Refractory hypertension and uncontrolled BP (SBP ≥ 140 and/or DBP ≥ 90) despite taking 3 or more antihypertensive drugs, including diuretics, were seen in 8 cases (1 aortitis case and 7 atherosclerotic stenosis cases) among 47 cases (17.0%, 3 of 50 missed BP or drug data from the outpatient clinic before PTRA). Causes of RAS were atherosclerosis (42 cases), FMD (6 cases), and Takayasu aortitis (2 cases), as shown in Table 1. One FMD case was complicated with Moyamoya disease (18-year-old male). The mean age was 61.5 years and mean BMI was 23.1. Twenty-one patients had DM and 8 had IGT. Twenty-seven patients had dyslipidemia. Twenty-one patients (42%) had bilateral RAS, and they were treated with one-step strategy. Mean BP at w0 was 152/80 mmHg, eGFR was 57.2 mL/min/1.73 m^2^, and other characteristics and subgroup characteristics divided by causes of RAS are shown in Table 1. Patients with atherosclerotic lesions were older and had a higher rate of coexisting DM and dyslipidemia. Among all patients, 6 were missing w1 BP measurements and 6 were missing y1 S-Cre measurements. The number of patients with an RI of ≥0.80 was only 3. There was no significant relationship between stenosis rate and RI (data not shown).

**Figure 1:**
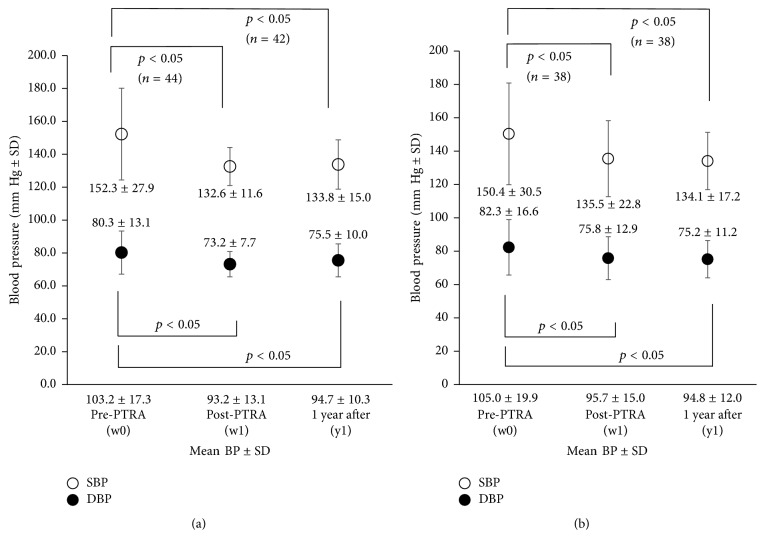
Change in blood ressure. (a) All cases. (b) Atherosclerotic RAS only. SBP and DBP were significantly reduced after PTRA, and the BP lowering effects continued until 1 year after. RAS: renal artery stenosis; BP: blood pressure; SBP: systolic BP; DBP: diastolic BP; PTRA: percutaneous transluminal renal angioplasty; SD: standard deviation.

SBP and DBP were significantly reduced from 152.3/80.3 mmHg to 132.6/73.2 mmHg (Figure 1(a)) after PTRA, and the BP lowering effects continued until 1 year after. The number of drugs patients had taken was significantly reduced from 1.98 to 1.28 (*p* < 0.05, *n* = 47) at the period of 1-2 weeks after PTRA, but the reduction did not last until 1 year after PTRA (1.95*p* = 0.39*n* = 42). Mean BP reduction of 5 mmHg or more was observed in 29 of 44 patients (66%), and reduced dose of antihypertensive drugs was observed in 23 of 50 (46%). Successful BP reduction was observed in 35 of 48 patients (73%). Limited to cases of atherosclerotic RAS, the reduction in BP remained significant (Figure 1(b)) and the successful BP reduction rate was similar to that of all cases (70% 28/40).

Univariate analysis of predictive and confounding factors for successful BP reduction are shown in Table 2. No items indicated a significant correlation with successful BP reduction.

**Figure 2:**
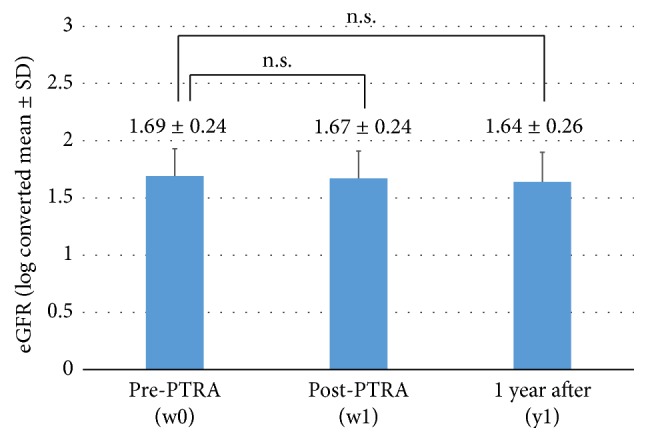
Change in eGFR. eGFR was not improved after PTRA. PTRA: percutaneous transluminal renal angioplasty; eGFR: estimated glomerular filtration rate; SD: standard deviation. ^*∗*^Paired *t*-tests were performed after log conversion of the values.

The changes in average eGFR throughout pre- and post-PTRA and 1 year after are shown in Figure 2. The estimated renal function neither improved nor worsened. Acute renal dysfunction, defined as an S-Cre rise of 0.5 mg/dL or more, was observed in 2 patients with atherosclerotic RAS and type 2 DM. Both patients recovered.

**Figure 3:**
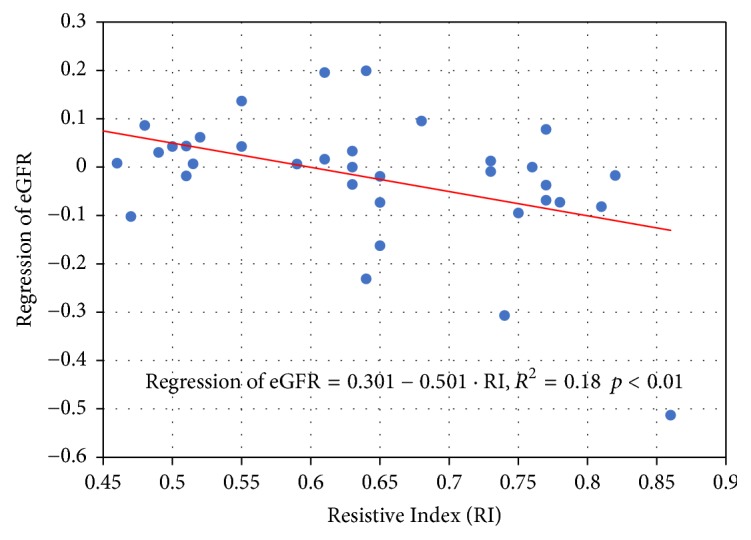
RI and regression of eGFR. Baseline RI was significantly correlated with deterioration of renal function. RI: resistive index. eGFR: estimated glomerular filtration rate. Regression of eGFR was calculated as log* *(eGFR y1) − log*  *(eGFR y0).

Renal function was “not changed or improved” in 23 of 44 patients (52%) at y1. Univariate analysis of confounding factors for regression of renal function is shown in Table 3(a). Baseline RI was significantly correlated with deterioration of renal function (Figure 3). Presence of “IGT or DM” tended to correlate with deterioration of renal function at y1 (Student's *t*-test, *p* = 0.05). There was a significant correlation between presence of “IGT or DM” and baseline RI (without IGT or DM 0.58 +/− 0.09, with IGT or DM 0.71 +/− 0.11, *p* < 0.01). We performed multivariate analysis for regression of renal function accounting for age, sex, BMI, baseline RI, and the dose of contrast medium. Baseline RI remained significant for the regression of renal function (Table 4(a)). In the subgroup of atherosclerotic RAS, lower RI showed a significant relationship with regression of renal function after correction for age, sex, BMI, and the dose of contrast medium (Tables 3(b) and 4(b)).

## 4. Discussion

In the present study, BP was improved in approximately 70% of patients, but the average eGFR remained unchanged from the baseline. We also demonstrated that patients with a higher RI were associated with a poor renal course, as reported by Radermacher et al. [18]. Higher RI is correlated with low eGFR and may be a consequence of progressed microangiopathy of the kidneys, which causes poorer renal prognosis.

We also demonstrated that patients with glucose intolerance tended to have worsened renal function. Hyperglycemia causes increased glomerular vascular resistance [21]. RI is reported to be higher in patients with DM than in the control [22]. Our data were in accordance with previous reports.

Although prognosis of RAS is known to be different between atherosclerosis and FMD [4], RI demonstrated a significant relationship with regression of renal function in all atherosclerosis patients in the present study. RI seems to be an important prognostic factor.

On the other hand, 12 subjects in the present study lacked RI measurements. The main reasons why the data were unavailable were undetectable blood flow signals in the kidneys due to severe renal damage or marked stenosis of the renal arteries. There were no significant differences in eGFR values between the subjects with available RI measurements and the subjects for which we could not obtain the RI value. Prognostic factors among such patients remain unclear in the present study.

Indication for PTRA in patients with RAS continues to be still controversial because the results of ASTRAL and CORAL failed to demonstrate beneficial effects of PTRA. It is possible that patients who may have benefited from PTRA were excluded and/or patients with mild stenosis were included in these studies.

Although our subjects, who were expected to have better outcomes than the subjects of ASTRAL or CORAL, demonstrated hemodynamically significant RAS, overall regression of renal function was not observed. In the results of ASTRAL, the decline in renal function over time was slightly slower in the revascularization group, but the difference was not significant after 34 months of follow-up [7, 13]. Renal function in our subjects was preserved for 1 year after PTRA. Therefore, a longer follow-up period seems to be needed to demonstrate the renoprotective effects of PTRA. According to the current guidelines [11–15], PTRA is recommended for patients with flash pulmonary edema, rapidly declining renal function and refractory hypertension, accelerated hypertension, unexplained unilateral small kidney, or renal arteries affected in the bilateral or single-functioning kidney. Both of the studies were criticized for the low applicability of PTRA for their included subjects.

It has been reported that approximately 20–45% of patients with atherosclerotic RAS are affected in the bilateral or single-functioning kidney [7, 23, 24]. In our study, patients with bilateral RA stenosis were observed in a similar proportion (Table 1). Although the results of the RCTs were negative, PTRA may be effective in limited high-risk cases [11, 12, 14]. However, our study could not demonstrate a correlation between bilateral RAS and improvement of renal function. Our study did not confirm the validity of the current recommendations, mainly due to too few cases and large heterogeneity between cases. On the other hand, the current recommendations for PTRA were mainly based on observational studies or consensus of the specialists [11–15]. Evidence based on the randomized trials is still very limited.

The limitations of our study are that it was a retrospective and single center design, with a limited number of subjects, which could not detect the difference in clinical course between causes of the RAS, and lack of control groups. Although we applied PTRA for patients with hemodynamically significant RAS, our criteria for applicability of PTRA were not strict, and therapeutic methods were at each doctor‘s discretion in each clinical situation. Furthermore, although the smoking habits of patients seem to be one of the determining factors of clinical outcome [25], we were unable to obtain the data in most cases due to a lack of detailed smoking history records.

## 5. Conclusion

In cases with hemodynamically significant RAS, PTRA was able to lower BP but was not effective in improving renal function.

Higher baseline RI demonstrated a significant correlation with poor renal outcome after PTRA. The presence of IGT or DM may be associated with poor renal outcome. Caution should be taken when PTRA for RVHT is considered in such patients.

## Figures and Tables

**Table 1:** Baseline characteristics. Data are described as “mean ± standard deviation,” or “number (percentage).” M: male; F: female; BMI: body mass index; IGT: impaired glucose tolerance; DM: diabetes mellitus; PSV: peak systolic velocity; RA: renal arteries; RI: resistive index; SBP: systolic blood pressure; DBP: diastolic BP; S-Cre: serum creatinine; eGFR: estimated glomerular filtration rate; proteinuria: patients who have uric protein ≥ ± or urinary albumin/creatinine ratio ≥ 30; chronic kidney disease: patients who have albuminuria or reduced eGFR (<60 mL/min/1.73 m^2^). ^*∗*^Missing data in one of two cases with aortitis. ^*∗∗*^The volume of iopamidol (300 mg iodine/mL).

	Atherosclerosis	Fibromuscular dysplasia	Aortitis	All subjects
Number (M/F)	42 (34/8)	6 (1/5)	2 (2/0)	50 (37/13)
Age (year)	68.0 ± 7.8	27.0 ± 11.2	28.5 ± 16.3	61.5 ± 17.2
BMI (kg/m^2^)	23.3 ± 3.5	22.3 ± 0 2.2	21.4 ± 1.2	23.1 ± 3.3
IGT or DM (%)	21 (50)	0 (0)	0 (0)	21 (42)
Dyslipidemia (%)	27 (64)	1 (17)	0 (0)	28 (56)
Calcium channel blockers (%)	33 (79)	3 (50)	2 (100)	38 (76)
ACE inhibitor (%)	6 (14)	0 (0)	0 (0)	6 (12)
Angiotensin II type 1 receptor blocker (%)	12 (29)	1 (17)	0 (0)	13 (26)
Diuretics (%)	13 (31)	1 (17)	1 (50)	15 (30)
*β*-Blockers (%)	14 (42)	2 (33)	2 (100)	18 (36)
*α*-Blockers (%)	4 (10)	0 (0)	0 (0)	4 (8)
Stenosis rate (%)	(82)	(81)	(83)	(82)
Bilateral stenosis (%)	18 (43)	1 (17)	2 (100)	21 (42)
PSV of the affected RA (m/sec)	2.4 ± 0.9	2.3 ± 1.4	5.4 ± 2.4	2.5 ± 1.2
Renal/aorta ratio	2.6 ± 1.0	2.9 ± 1.4	4.1 ± 1.0	2.7 ± 0.9
RI (poststenotic site)	0.66 ± 0.10	0.47 ± 0.1	0.52^*∗*^	0.63 ± 0.12
PRA (ng/mL/hr)	4.7 ± 6.1	7.3 ± 7.5	4.2 ± 1.4	5.0 ± 6.1
PRA (log converted)	0.41 ± 0.51	0.71 ± 0.37	0.65 ± 0.15	0.45 ± 0.49
PAC (ng/dL)	16.6 ± 10.0	35.5 ± 49.8	14.2 ± 2.1	18.8 ± 19.4
SBP (mmHg)	153 ± 30	151 ± 8	159 ± 27	152 ± 28
DBP (mmHg)	80 ± 14	84 ± 8	86 ± 8	80 ± 13
Pulse Rate (/min)	67 ± 7	68 ± 7	72 ± 3	67 ± 7
S-Cre (mg/dL)	1.39 ± 0.66	0.59 ± 0.13	0.84 ± 0.23	1.28 ± 0.67
eGFR (mL/min/1.73 m^2^)	47.2 ± 23.2	113.6 ± 34.7	99.2 ± 45.2	57.2 ± 34.1
eGFR (log converted)	1.63 ± 0.20	2.04 ± 0.13	1.97 ± 0.20	1.69 ± 0.24
Proteinuria (%)	24 (60) *n* = 40	2 (40) *n* = 5	0 (0)	26 (55) *n* = 47
eGFR < 60 mL/min/1.73 m^2^ (%)	31 (78) *n* = 40	0 (0) *n* = 5	0 (0)	31 (66) *n* = 47
Chronic Kidney Disease (%)	36 (88) *n* = 41	2 (40) *n* = 5	0 (0)	38 (79) *n* = 48
Dose of Contrast Medium (mL)^*∗∗*^	107.6 ± 41.4	102.3 ± 19.4	120^*∗*^	107.2 ± 38.7

**Table 2:** Univariate analysis of predictive and confounding factors for successful reduction of blood pressure. Successful reduction of BP was defined as reduction in mean BP of 5 mmHg or more, or reduction of antihypertensive dose. ^*∗*^Logistic regression analysis, other parameters were analyzed with the chi-square test. ^!^Plasma renin activity and eGFR were analyzed after log conversion. BP: blood pressure; BMI: body mass index; IGT: impaired glucose tolerance; DM: diabetes mellitus; eGFR: estimated glomerular filtration rate.

	Number	Odds ratio	*p* value
Age (years)^*∗*^	48	5.14	0.25
Sex (female)	48	1.33	0.70
BMI (kg/m^2^)^*∗*^	48	4.61	0.37
IGT or DM	47	0.23	0.09
Dyslipidemia	47	0.59	0.45
Bilateral renal artery stenosis	48	1.08	0.91
Resistive index^*∗*^	38	0.51	0.30
Plasma renin activity^*∗*!^	48	0.22	0.29
Mean BP (mmHg)^*∗*^	47	0.04	0.16
eGFR^*∗*!^	45	0.20	0.24

**(a):** 

	Number	Mean ± SD		Gradient	*R* ^2^	*p* value
Age (years)^*∗*^	44			0.00	0.02	0.40
Sex	44	male −0.013 ± 0.118	female −0.058 ± 0.170			0.32
BMI (kg/m^2^)^*∗*^	44			0.01	0.23	0.32
IGT or DM	43	Normal GT 0.01 ± 0.02	IGT or DM −0.07 ± 0.03			0.05
Dyslipidemia	43	Dyslipidemia − −0.06 ± 0.03	Dyslipidemia + 0 ± 0.03			0.15
Bilateral renal artery stenosis	44	Unilateral −0.02 ± 0.03	Bilateral −0.03 ± 0.03			0.66
Baseline RI^*∗*^	35			−0.50	0.19	<0.05
Plasma renin activity^*∗*#^	44			0.07	0.05	0.14
Mean blood pressure (mmHg)^*∗*^	44			0.00	0.07	0.09
eGFR^*∗*#^	44			−0.08	0.02	0.34
Dose of contrast medium (mL)	47			0.00	0.01	0.55

**(b):** 

	Number	Mean ± SD		Gradient	*R* ^2^	*p* value
Age (years)^*∗*^	37			0.00	0.00	0.32
Sex	37	male −0.016 ± 0.123	female −0.081 ± 0.204			0.13
BMI (kg/m^2^)^*∗*^	37			0.01	0.01	0.27
IGT or DM	36	Normal GT 0.02 ± 0.13	IGT or DM −0.07 ± 0.15			<0.05
Dyslipidemia	36	Dyslipidemia − −0.08 ± 0.04	Dyslipidemia + 0.00 ± 0.02			0.05
Bilateral renal artery stenosis	37	Unilateral −0.02 ± 0.02	Bilateral −0.03 ± 0.01			0.35
Baseline RI^*∗*^	30			−0.66	0.23	<0.05
Plasma renin activity^*∗*#^	37			0.07	0.03	0.16
Mean BP (mmHg)^*∗*^	34			0.00	0.05	0.10
eGFR^*∗*#^	37			−0.17	0.03	0.14
Dose of contrast medium (mL)	35			0.00	0.01	0.52

**(a):** 

	Parameter estimate	*t* value	*p* value
Age (years)	0.00	0.52	0.61
Sex (female)	0.05	2.18	<0.05
BMI (kg/m^2^)	0.01	1.81	0.08
Baseline RI	0.72	−3.12	<0.05
Dose of contrast medium	0.00	−1.11	0.28

**(b):** 

	Parameter estimate	*t* value	*P* value
Age (years)	0.00	−0.13	0.90
Sex (female)	0.05	1.73	0.10
BMI (kg/m^2^)	0.01	1.73	0.09
Baseline RI	0.69	−2.65	<0.05
Dose of contrast medium	0.00	−1.18	0.25
